# The History of a Royal Fistule from *La France Medicale*

**Published:** 1874-05-01

**Authors:** 


### THE HISTORY OF A ROYAL FISTULE.

*Translated for* THE EXAMINER, *from La France Medicale, March* 4, 1874.

LOUIS XIV., at forty-seven years of age, had attained the apogee of his glory. He had built Versailles, Trianon and Marly; had enlarged greatly the palaces of Fontainebleau, Saint Germain, Saint Cloud and Chambord; had received from the authorities of Paris the title of "The Great;" his life had become much more regular, and he had abandoned the last of his mistresses, Mlle. de Fontanges. Maria Theresa died in 1683, and he subsequently contracted a secret marriage with the widow of Scarron, Mme. de Maintenon.

January 15th, 1686, the king complained of a small tumor in the perineum, on one side of the raphe, two fingers'-breadths from the anus, barely sensitive to the touch, and destitute of pain, redness and pulsation. It did not interfere with defecation, nor exercise on horseback, but subsequently increased somewhat in size, and became so indurated, that on the 31st of January, it was determined to attempt its resolution.

On the 5th of February, Daquin, the king's chief physician, prescribed a poultice, composed of beans, broom-rape, barley, rye and flaxseed soaked in wine and vinegar, which was to be renewed every five or six hours. During the next few days, in which the king was confined to his bed, a plaster of white lead (prepared by boiling) and hemlock, was also applied. The pain of the swelling increased to such an extent, that by February 16th the patient was obliged to retain the recumbent position. The skin meantime had become somewhat reddened in the vicinity of the little tumor, which had not, however, increased in size. It was thought that suppuration had occurred, and, therefore, at the point where ulceration seemed imminent, an ointment of colophony, yellow wax, resin and olive oil was applied, and the whole covered with a plaster containing yellow wax, turpentine, Burgundy pitch, and the acetate of copper. The desired effect was produced. Fluctuation became evident on the 18th, and the pain increased, as in ordinary suppuration.

The courtiers, as usual, interested themselves greatly in the malady of the monarch, and were loud in praise of a certain sparadrap, with which the inventress, Mme. de La Daubiere, had accomplished marvellous results. The physicians consented to its use, on condition that its composition should be made known to them, and that it should be prepared by the royal apothecaries. The formula was as follows:

Gum elemi and turpentine, boiled in plantain water, } lb. ss.

Yellow wax, ℥ viij.

Balsam of Peru, ℥ iss.

No results were obtained by the use of this compound, so the former treatment was renewed; and on the evening of February 19th the abscess burst, and discharged during the ensuing night. A slightly indurated projection was still evident after this occurrence.

On the 20th of February caustic was added to the ointment of colophony, which had the effect of enlarging the wound. It then gave issue to a thicker and more sanguinolent pus, which escaped incessantly, but brought about a notable diminution in the size of the tumor.

On the 21st of February the king suffered from an attack of gout in the right foot, and on the following day from lassitude and cephalalgia; but there was no fever. The orifice of the sinus slightly contracted; there was a reddish, sero-sanguinolent discharge; no insomnia. On the 23rd of February it was determined to open the abscess from below. Two cauteries were applied, an eschar made, and the opening effected with a lancet. Pus escaped, and the early dressings were renewed. There was some gouty pain during the night. On the 24th the abscess became indurated, and resolution was attempted by introducing into the wound a tent, smeared with a balsam composed of the oils of flaxseed, juniper, olives, cloves, laurel, and turpentine, with aloes, sulphate of zinc and acetate of copper. There was some sleep at night, and less gouty pain. On the 27th of February the pus became more thick, and fomentations were applied, consisting of the decoction of absinthe, roses, pomegranate rind, and leaves of myrrh in red wine. February 28th, the balsam was discontinued, and the abscess injected with a lotion for wounds.

March 2nd: There was no resolution of the tumor. It was dressed with red precipitate, one drachm, and the colophony ointment, one-half pound. The sleep of the patient was disturbed, and the gout increased in severity, yielding, however, to treatment by the 8th of March.

The disease went on, alternately progressing and retrograding, until the middle of May. The king had had hitherto an external blind fistula; but, May 17th, when injecting the cavity, it was noticed that the injected fluid did not completely return, which led to the suspicion that the intestine had been injured, and the fistula had become complete—the ulcer sometimes appearing cured and sometimes re-opening. To remove all doubt on this point, an exceedingly red decoction of hypericum perforatum (St. John's Wort) was injected, which did not return by the wound; and the king, placing himself upon a chamber, passed the entire decoction from the bowel. Afterward, to make sure of the locality of the intestinal lesion, a probe was introduced through the fistula, and the index finger, when passed into the rectum, encountered the extremity of the probe, at a distance of about two or three fingers'-breadths above the anus. A small quantity of pus and blood followed its withdrawal.

On the 27th of May the king mounted his horse. In August he had a quartan fever, and was bled and purged. Cinchona was subsequently administered in the following manner: One ounce of cinchona in powder was infused in a pint of good Burgundy wine. During the first twenty-four hours the mixture was agitated several times, and finally left to stand. Of this, four to five ounces were administered every four hours, night and day. In a few days the patient was relieved.

But the fistula remained unchanged. It was evident that there was but one means whereby to cure it; and that was an operation. But, at the court of Louis XIV. it was not easy to induce submission to such procedures. All sorts of people still announced infallible cures; and a trial of these must of course be made. After the plasters and ointments, the waters of Bareges were vaunted. The rumor spread that the king was about to make trial of these waters; but it was considered desirable that they should be first tried on some of his subjects. Four persons affected with anal fistula were first sent to Bareges, under the direction of Louvois, surgeon-in-ordinary to the king. Lotions and injections of these waters were used ineffectually. Finally, a woman appeared who declared that she had been cured of a fistula by the waters of Bourbon-l'Archambault. Four additional patients were at once despatched to Bourbon, with a royal surgeon, and returned in the same condition. Lastly, beds were established under the superintendence of Louvois, and filled with patients affected with fistulæ. They were treated under the surveillance of the surgeon-in-chief, Felix, by those who pretended they could cure them. All failed.

The surgeon Bessieres, who had free license to speak at the court, declared to the king that all remedies were, and would be, valueless; and that no cure was possible, except by an operation. For a long time the surgeon-in-chief, Felix, had proposed this; but had expected that the royal patient would consent only after he had become thoroughly disgusted with all the measures proposed by the empirics.

Louis XIV. had left Fontainebleau and returned to Versailles, thoroughly resolved to undergo an operation at the hands of Felix, to whom he had given permission to choose his assistants. The king decided that the affair was to be kept secret.

On the 18th of November, 1686, about eight o'clock in the morning, Felix, having Bessieres for an assistant, proceeded to operate, in the presence of the physician-in-chief, Daquin; the physician-in-ordinary, Fagon; and Louvois. Taking in his left hand a bistoury, made expressly for the purpose, and having a probe attached to its extremity, he passed the latter into the rectum by the fistula. The finger of the right hand, pressed into the intestine, encountered the flexible probe, and withdrew it from the anus; thus allowing the bistoury to cut with great promptness and facility the tissues between the fistula and the gut. He finally introduced scissors into the fundus of the wound, and divided the intestine somewhat above the opening, as well as all bridles of tissues which he encountered. One hour after the operation the patient was bled from the arm, and placed upon a severe regimen—abstinence from all solid alimentation; a weak broth being only permitted morning and night.

If the chronicles of that period can be trusted, Felix, despite his skill, hesitated so long before making the first incision, that his royal patient found it necessary to re-assure him; and the result of this to the surgeon was that he retained, during the remainder of his life, the trembling which he then first experienced.

The bistoury employed upon this occasion is preserved in the Museum, by the Faculty of Medicine of Paris. Its form is exceedingly primitive. It consists of a blade somewhat more than one-third of an inch broad, and eight or ten times as long, slightly curved upon itself, and terminating in a flexible stylet.

Although the operation had been done with all the requisite skill, there remained in the site of the fistula, callosities which interfered with cicatrization. In order to their removal, the "suppurative" and resolvent ointments, as well as mercury, were tried to no purpose. Twenty-two days after the operation, Felix extirpated these growths with the knife.

December 27th, the wound was almost closed, and the dressing consisted only of charpie and the lotion for wounds. But there remained an indurated nodule in the neighborhood of the anus, interfering with cicatrization. On the 1st of January, 1687, Felix scarified this callosity deeply, and then applied to it red precipitate in powder, which produced a deep eschar. On the 7th of January there was a new and final scarification with lancet and scissors, the fifth since the beginning. An escharotic, compounded of equal parts of alum and red precipitate, was applied, and succeeded by excessive pain. Dysuria and a sanguinolent discharge occurred. The wound was bathed with barley-water; but the patient passed an uncomfortable night. From this date improvement was dedecided; the eschars separated, and cicatrization was complete by the 14th of January. February 18th the patient had another gouty attack; but on the 18th of March the king again mounted his horse.

It is curious to note the opinion of the physician-in-chief, Daquin, on the nature of this fistula. This is what he says:

"This tumour hath never been painfull, and hath displayed neither redness nor inflammation from its beginnynge throughout its progress. It was made to suppurate only with difficulty, and resolution could in no wise be obtayned. The greater parte thereof soon became indurated and scirrhous, which proveth it to be a tumour of humour melancholic, crude, cold and indigestive, such as those which habitually produce scirrhous growths. And, forasmuch as it seemed to be indolent, having but little salt and acrimony (though it was permitted to remain but for a short while, since it was opened a few days after its appearance) it is difficult to understand in what manner the gut was penetrated. To avoid coming to any false conclusions, it is better to believe that the rent occurred before the tumour appeared, and that the vessel charged with the infecting humour which produced it, entered the intestine from without, became involved in the folds of the anus, and traversed its membranes as far as the middle of the perineum," etc.

And these lines are signed by the name of Daquin! How well Moliere knew the physicians of his age!

Louis XIV. rewarded his physicians and surgeons with a princely hand. Felix had fifty thousand crowns; Daquin, a hundred thousand livres; Fagon, eighty thousand livres; Bessieres, forty thousand. The four apothecaries received each twelve thousand livres; and La Raye, the surgeon-in-chief's son, received four hundred pistoles, or forty thousand livres.

Although the operation upon anal fistula by incision had long been indicated, the idea of it had fallen into oblivion, in consequence of the fear of such serious accidents as hæmorrhage and incontinence of fæcés. And thus, those who were afflicted with this distressing infirmity, preferred to retain their fistula, rather than expose themselves to the danger of a worse accident. The fistula of Louis XIV. changed the face of affairs.

Celsus, who lived in the time of Augustus, describes the indications for this operation with great distinctness. (Book VII., chap. iv. 4.) He speaks of cutting the fistula by the aid of a thread, passed through the two openings; but he prefers the employment of the bistoury, especially when the fistule is complete. "*In haec genera demisso specillo, duabus lineis incidenda cutis est: et media inter eas habenula tenuis admodum ejiciatur, ne protinus oræ coeant.*" (In this variety a sound should be introduced; then the tissues incised for the space of two lines, and the little bridle removed which separates the walls of the fistula, and prevents their union.)

Felix practiced for several months before submitting his sovereign to the risks of an operation, a circumstance which, perhaps, explains the emotion he then experienced. He died May 25th, 1703, aged fifty years, rich and honored, but leaving no page of surgical record traced by his pen. He was succeeded by George Mareschal, surgeon to La Charite of Paris, who in 1696 had been already summoned in consultation to the bedside of the king, then affected with an abscess of the nucha. In 1701, Fagon, the physician-in-chief, old, asthmatic, deformed, and epileptic, was affected with stone in the bladder. He was successfully operated on by Mareschal, and received on that occasion from the king one hundred thousand francs. We are not informed as to the *honorarium* given to the operating surgeon; but two years after, on the death of Felix, it was Mareschal who was elevated to the rank of surgeon-in-chief to the king, a position retained by him under both Louis XIV. and Louis XV., while Fagon was passed over.

The history of medicine at this epoch clearly indicates the difference between the learning of physicians and surgeons, a difference greatly to the advantage of the latter. And yet it was at this very time that physicians were seeking to obtain a supremacy over the members of the College of Surgeons.

